# Review of Dengue Hemorrhagic Fever Fatal Cases Seen Among Adults: A Retrospective Study

**DOI:** 10.1371/journal.pntd.0002194

**Published:** 2013-05-02

**Authors:** Sing-Sin Sam, Sharifah Faridah Syed Omar, Boon-Teong Teoh, Juraina Abd-Jamil, Sazaly AbuBakar

**Affiliations:** 1 Tropical Infectious Diseases Research and Education Center (TIDREC), Department of Medical Microbiology, Faculty of Medicine, University of Malaya, Kuala Lumpur, Malaysia; 2 Infectious Diseases Unit, Faculty of Medicine, University of Malaya, Kuala Lumpur, Malaysia; Oxford University Clinical Research Unit, Viet Nam

## Abstract

**Background:**

Dengue is a mosquito-borne viral disease endemic in many countries in the tropics and sub-tropics. The disease affects mainly children, but in recent years it is becoming more of an adult disease. Malaysia experienced a large dengue outbreak in 2006 to 2007, involving mostly adults, with a high number of deaths.

**Methodology/Principal Findings:**

We undertook a retrospective study to examine dengue death cases in our hospital from June 2006 to October 2007 with a view to determine if there have been changes in the presentation of severe to fatal dengue. Nine of ten fatal cases involved adult females with a median age of 32 years. All had secondary dengue infection. The mean duration of illness prior to hospitalization was 4.7 days and deaths occurred at an average of 2.4 days post-admission. Gastrointestinal pain, vomiting, diarrhea, intravascular leakages and bleeding occurred in the majority of cases. DSS complicated with severe bleeding, multi-organ failure and coagulopathy were the primary causes of deaths. Seven patients presented with thrombocytopenia and hypoalbuminemia, five of which had hemoconcentration and increased ALT and AST indicative of liver damage. Co-morbidities particularly diabetes mellitus was common in our cohort. Prominent unusual presentations included acute renal failure, acute respiratory distress syndrome, myocarditis with pericarditis, and hemorrhages over the brain and heart.

**Conclusions:**

In our cohort, dengue fatalities are seen primarily in adult females with secondary dengue infection. The majority of the patients presented with common clinical and laboratory warning signs of severe dengue. Underlying co-morbidities may contribute to the rapid clinical deterioration in severe dengue. The uncommon presentations of dengue are likely a reflection of the changing demographics where adults are now more likely to contract dengue in dengue endemic regions.

## Author Summary

Dengue continues to be a major mosquito-borne disease of serious public health concern. Children are usually the most affected group, but in recent decades, dengue and severe dengue have become more common among adults. Here we reviewed ten fatal dengue cases with a view to determine if there have been changes in the presentation of severe to fatal dengue. Our findings revealed high dengue mortality among adult females, associated with secondary dengue infection. Underlying co-morbidities particularly diabetes mellitus were common in our cohort. This may contribute to the rapid deterioration of clinical condition seen in severe dengue. Most patients presented with common clinical and laboratory warning signs for severe dengue. Uncommon presentations seen among our fatal cases are likely a reflection of the changing demography of dengue from children to more of an adult disease in dengue endemic countries. Our findings, hence, emphasize the importance of healthcare awareness of clinical warning signs for severe dengue, especially in adult females with underlying co-morbidities and secondary dengue infection.

## Introduction

Dengue virus (DENV) infection is a global health threat affecting at least 3.6 billion people living in more than 125 countries in the tropics and subtropics [1]. It is among the most important arthropod-borne diseases. All four dengue virus serotypes (DENV-1, DENV-2, DENV-3 and DENV-4) can cause dengue. The disease can present as a mild self-limiting illness, dengue fever (DF), or as the more severe forms of the disease, dengue hemorrhagic fever (DHF) and dengue shock syndrome (DSS) [2]. The World Health Organization (WHO) 2009 guidelines classify patients into three groups; dengue without warning signs, dengue with warning signs and severe dengue [3]. Clinical manifestation of severe dengue includes severe bleeding, severe organ involvement and severe plasma leakage. Most dengue deaths are associated with DHF/DSS (WHO 1997 guidelines) and severe dengue (WHO 2009 guidelines).

DF and DHF were first documented in Malaysia in 1902 and 1962, respectively [4], [5]. A major dengue epidemic was recorded in 1973, and since then dengue has become endemic in Malaysia with major outbreaks occurring every 3–4 years [6], [7]. There were a number of reports describing the clinical features and risk factors associated with the severe manifestations of dengue and dengue-related deaths during the first two decades following the 1973 epidemic. During this period, children were the most predominant group affected, hence contributed substantially to the clinical description of severe dengue [8], [9], [10]. In the last two decades, however, the number of dengue cases had escalated exponentially. There were 48,846 cases and 98 deaths in 2007 in Malaysia with those aged 15–35 years old contributing to at least 48% of the total number of dengue cases [11]. The trend of higher percentage of adults contracting dengue has also been reported in other dengue endemic countries [12]. This review of fatal cases of dengue infection was undertaken in light of this changing epidemiology of dengue in Malaysia and in this region.

## Methods

### Ethics Statement

The study was approved by the University Malaya Medical Center (UMMC) Medical Ethics Committee (ethics committee/IRB reference number: 611.10). Informed consents were not obtained from the patients as this was a retrospective study.

### Clinical Case Definition

The medical records of ten patients at UMMC with dengue-related deaths during the period from June 2006–October 2007 were reviewed and notes were transcribed into standardized data entry forms. Disease severity was classified following the WHO 1997 guideline [2]. This was done as the clinical notes were all in accordance to the WHO 1997 guidelines.

### Serology

The acute-phase serum samples were obtained from the UMMC Diagnostic Virology Laboratory Repository. Convalescent serum sample was available for only one of the patients. Serum samples were respectively tested for dengue-specific IgM and IgG antibodies using SD Dengue IgM and IgG Capture ELISA kits (Standard Diagnostics, Korea) [13]. Serum samples were also tested for dengue-specific NS1 antigen using both pan-E Early Dengue ELISA kit (Panbio, Australia) and Platelia Dengue NS1 Ag assay (Bio-Rad Laboratories, USA).

### Virus Isolation and Genotyping

Virus isolation was performed by inoculating the serum samples onto monolayer of *Aedes albopictus* C6/36 cells in 24-well plate. The cells were maintained in EMEM supplemented with 2% fetal bovine serum at 28°C for one week. RNA was extracted from cell culture supernatant using QIAamp Viral RNA Mini Kit (Qiagen, Germany). Genotyping was done using the in-house-developed multiplex RT-PCR genotyping kit which amplified a portion of viral NS3 gene. Amplification was performed as previously described [14], [15].

## Results

There was a dramatic increase in the number of dengue cases seen at the UMMC in Malaysia from 200 cases in the year 2000 to 1,826 and 2,096 dengue cases in 2006 and 2007, respectively. There were ten fatal dengue cases between the periods of June 2006 to October 2007. The demographics of all fatal cases are illustrated in Table 1. Nine cases were female. The age range was between 11 to 59 years with a median age of 32 years. Adults over 18 years old comprised 80% of all the fatal cases. Of the 10 fatal cases, four were Malay, three Indian and two Chinese. One patient was a foreigner from Bangladesh. Three of the patients were known diabetics.

**Table 1:** Demographics, co-morbidities, clinical features and postmortem findings of fatal dengue seen at UMMC between June 2006 to October 2007.

Patient	Age/Sex^a^/Race^b^	Duration of illness prior admission/Duration of hospitalization (day)	Co-morbidities	Bleeding Manifestation^c^	Postmortem	Cause of death^d^ (as reviewed by the authors)
**1**	12/F/C	Unknown/0	-	-	petechiae at pleural surfaces, lung congestion and edema, large and granular liver, kidney with congested medulla, enlarged spleen; Histology: pulmonary hypertension (lungs) and severe fatty change in liver	Unascertained; Postmortem: pulmonary hypertension due to chronic obstructive airway disease, obesity
**2**	19/F/M	9/0	-	-	-	DHF/DF
**3**	40/M/B	3/1	newly diagnosed DM	petechiae	-	DSS
**4**	34/F/M	3/1	newly diagnosed DM; adrenal adenoma	petechiae, needle insertion site, RT, PV,PR	hemorrhage over hemispheres (brain) and septum (heart), pleural effusion and lung congestion, blood in stomach	DSS with ARF and severe bleeding
**5**	39/F/M	4/1	-	petechiae, PR, PV, blood in NG tube, hematoma	-	DSS, DIC, severe bleeding, MOF (ARF, ALF)
**6**	42/F/I	5/1	DM type 2	-	pericardial and pleural effusion, hemorrhage over endocardium, fluid in peritoneal cavity and stomach, enlarged liver and spleen; Histology: severe lung congestion with intra-alveolar hemorrhage, spleen congestion, severe fatty infiltration in heart (suspecting of arrhythmogenic dysplasia), liver fatty change	DSS and severe bleeding
**7**	18/F/I	6/3	-	petechiae	-	DSS with MOF (ARF, ARDS), coagulopathy and severe bleeding
**8**	59/F/C	5/3	-	PR	-	DSS complicated with cardiogenic shock secondary to myocarditis and pericarditis
**9**	11/F/I	3/4	-	gums, blood stained ETT suctioning and coffee ground aspirate in NG tube	-	DSS. APO during reabsorption phase. Septiceamic shock secondary to presumed HAP (Blood & BAL culture negative; severe lactic and metabolic acidosis). New CXR change.
**10**	30/F/M	4/5	-	petechiae, GI, PV, PR, gum, epistaxis	-	DSS with severe GI bleeding, MOF, DIC

a Sex- Female (F); Male (M).

b Ethnicity- Malay (M); Chinese (C); Indian (I); Others, Bangladesh (B).

c Bleeding Site- Bleeding per vaginum (PV); Bleeding per rectum (PR); Gastrointestinal bleeding (GI); Respiratory tract (RT); Endotracheal tube (ETT); Nasogastric (NG).

d Cause of death- Acute liver failure (ALF); Acute pulmonary oedema (APO); Acute renal failure (ARF); Multi-organ failure (MOF); Disseminated intravascular coagulation (DIC); Acute respiratory distress syndrome (ARDS); Hospital acquired pneumonia (HAP); Bronchoalveolar lavage (BAL); Chest X-ray (CXR).

Two patients (Patient 1 & Patient 2) were brought in dead to the hospital (dead on arrival, DOA); whereas the remaining patients succumbed to the infection within 1–5 days of admission (an average of 2.4 days). Four out of the eight admitted patients had rapid deterioration of their clinical features and died within 24 hours of admission. The mean duration of illness prior to hospitalization was 4.7 days.

There was no clinical history related to dengue fever present for Patient 1. She had collapsed and became unresponsive while playing at home. Patient 2 was seen at the outpatient clinic four days before she died with a diagnosis of probable dengue fever. Clinical presentations of nine patients (Patients 2–10) are summarized in Table 2. Persistent vomiting (n = 9), body ache (n = 8), bleeding (n = 7), plasma leakage (n = 7), abdominal pain (n = 6), diarrhea (n = 6) and dehydration (n = 6) were the commonest clinical presentations. All nine patients had a history of fever with or without chills and rigors prior to admission. However, only five had a recorded temperature of >37.5°C on admission, with only three recording a temperature of >38°C. One patient was hypothermic (35.5°C) on admission. Plasma leakage (presence of pleural effusion, ascites or pericardial effusion) was noted in seven patients, including in Patient 6 where the presence of effusion was seen on postmortem (Table 1).

**Table 2:** Summary of clinical presentation in dengue fatal cases seen at UMMC between June 2006 to October 2007.

	Patient									Frequency
Clinical Features	2^a^	3^b^	4	5	6	7	8	9^c^	10	No. (%)
**Symptoms**										
History of Fever/Chills/Rigors	+	+	+	+	+	+	+	+	+	9 (100)
Headache		+				+		+	+	4 (44)
Abdominal pain		+	+			+	+	+	+	6 (67)
Rash		+				+			+	3 (33)
Bodyache/Myalgia	+	+	+	+		+	+	+	+	8 (89)
Arthralgia	+					+		+		3 (33)
Vomit/Nausea	+	+	+	+	+	+	+	+	+	9 (100)
Diarrhea		+	+		+	+		+	+	6 (67)
Bleeding (Petechiae, Gum, GI, PR, PV, etc)		+	+	+	+^1^	+	+	+	+	8 (89)
Lethargy						+	+	+		3 (33)
Giddiness						+	+		+	3 (33)
Faint						+				1 (11)
Confused		+								1 (11)
Restless		+				+	+	+	+	5 (56)
Shortness of breath		+		+	+				+	4 (44)
**Signs**										
Oliguria				+		+	+	+	+	5 (56)
Anuria		+		+					+	3 (33)
Dehydration		+	+			+	+	+	+	6 (67)
Ascites				+	+^1^		+	+	+	5 (56)
Pleural effusion		+	+	+	+^1^		+	+	+	7 (78)
Pericardial effusion			+		+^1^		+			3 (33)
Liver enlargement			+		+^1^			+		3 (33)
Tachycardia		+		+	+	+	+	+	+	7 (78)
Tachypnoe				+		+	+	+		4 (44)
Shock		+	+	+		+	+	+	+	7 (78)

a Brought in dead;

b Male;

c Child.

1 Autopsy.

Bleeding per vaginum (PV); Bleeding per rectum (PR); Gastrointestinal bleeding (GI).

Hematological tests were not available for Patient 1 and 2, and limited results were available for Patient 6. Severe thrombocytopenia (<50×10^9^/L) and hypoalbuminemia (≤16 g/L) were seen in seven patients. An increased alanine aminotransferase (ALT) and aspartate aminotransferase (AST) of more than 1000 IU/L were found in five patients, and hemoconcentration in five cases (Table 3). Coagulation profiles of seven patients showed prolonged activated partial thromboplastin time (APTT), ranging from 45.1 to >200 seconds.

**Table 3:** Laboratory diagnosis and hematological findings of samples from fatal dengue cases seen at UMMC between June 2006 to October 2007.

Laboratory findings	Patient 1	Patient 2	Patient 3	Patient 4	Patient 5	Patient 6	Patient 7	Patient 8	Patient 9	Patient 10
**Virus Isolation**	−	N/A	−	−	−	−	N/A	−	DENV1	−
**NS1 antigen**	+	N/A	+	+	+	+	N/A	+	+	+
**Dengue IgM**	+	+	−	−	−	+	+	+	+	+
**Dengue IgG**	+	+	+	+	+	+	+	+	+	+
**Hemoconcentration (hematocrit >20%)**	N/A	N/A	−	−	+	+	−	+	+	+
**Thrombocytopenia (<50 × 10^9^/L)**	N/A	N/A	+	+	+	N/A	+	+	+	+
**Prolonged APTT (>38.9 secs)**	N/A	N/A	+	+	+	N/A	+	+	+	+
**Prolonged TT (>19 secs)**	N/A	N/A	−	+	+	N/A	−	+	+	+
**Elevated AST (>1000 IU/L)**	N/A	N/A	−	+	+	N/A	+	+	−	+
**Elevated ALT (>1000 IU/L)**	N/A	N/A	−	+	+	N/A	+	+	−	+
**Increased Total Bilirubin (>17 µmol/L)**	N/A	N/A	+	+	+	N/A	+	+	+	+
**Hypoalbuminemia (<35 g/L)**	N/A	N/A	+	+	+	N/A	+	+	+	+

‘+’ = Positive; ‘−’ = Negative; N/A = Not available.

Activated partial thromboplastin time (APTT); Thrombin time (TT); Alanine aminotransferase (ALT); Aspartate aminotransferase (AST).

All, except Patient 1 and Patient 2, experienced rapid clinical deterioration following admission and were transferred to the intensive care units (ICU). The diagnosis of dengue was not apparent in Patient 1 even after a postmortem. A postmortem was not performed for Patient 2. All eight remaining fatal cases had a clear diagnosis of DSS (Table 1). Five patients had severe bleeding, either from gastrointestinal tract or from per vaginal bleeding. The postmortem showed hemorrhage in the brain and heart as well. One patient had myocarditis and pericarditis, which led to cardiogenic shock refractory to fluid resuscitation. One patient developed acute pulmonary oedema during the reabsorption phase and subsequently developed septiceamic shock secondary to hospital acquired pneumonia (Table 1). Her blood and bronchoalveolar lavage culture was negative. The patients who presented with DSS were resuscitated with intravenous fluid. Blood products used included whole blood, platelet concentrate, fresh frozen plasma, and cryoprecipitate. Six patients received blood product transfusion.

Post-mortem examination was performed in three patients (Patient 1, 4 and 6) (Table 1). The postmortem results in Patient 4 and Patient 6 were supportive of DSS. The cause of death for Patient 1 remained uncertain despite postmortem examination. The postmortem suggested pulmonary hypertension secondary to chronic obstructive airway disease and obesity as the possible cause of death.

Dengue infection was confirmed in all patients by dengue serological tests. Seven were both IgM and IgG positive while three were IgM negative and IgG positive. Eight of the patients (two were unavailable) tested positive for dengue NSI antigen with DENV-1 isolated from one case. The presence of anti-dengue IgG antibody concurrent with positive detection of dengue NS1 antigen confirmed secondary infections in eight patients. Of the eight patients, anti-dengue IgG titres in five patients were higher than IgM titres. Dengue NS1 antigen test was not performed in two cases (Patient 2 and 7). However, the presence of IgG in their serum within a period of less than two weeks since the onset of illness suggests secondary infection.

## Discussion

The present study reviewed the clinical features of ten fatal cases of DHF/DSS seen at UMMC, a major teaching and referral hospital, during the period when Malaysia experienced dramatic increase in dengue cases. The dengue deaths were seen primarily in adult females and were associated with secondary dengue infection. Majority of the patients presented with common clinical and laboratory warning signs of severe dengue before death. Underlying co-morbidities may be the contributing factors towards the rapid deterioration in severe dengue. Other complications included involvement of other organs including the brain, heart, liver and kidneys. This is reflective of the shift in the demographics of dengue cases in Malaysia where more adults are affected.

In Southeast Asia, severe and fatal dengue has been primarily described among children. Similar pattern was also observed in Malaysia until early 1982 [16], [17], [18], [19] where the percentage of dengue cases became most common among adults of 13 to 35 years old. In UMMC, the median age of laboratory-confirmed dengue cases between the year 2006 to 2007 was 25 years (age range 1 month to 88 years). The majority of cases were adults of 21 to 25 years and >35 years old, with mean percentages of 20.5% and 23%, respectively (unpublished data). This trend is similar in most dengue endemic countries in Southeast Asia [20], [21]. With this changing demography, it is possible that there are features of severe dengue leading to death that could be different from those seen in children.

In the present review, fatal cases comprised mainly of adults of >18 years old. There is a higher preponderance of fatal DHF/DSS amongst females. This is despite >55% of dengue cases seen at UMMC between the year 2006–2007 occurred in males (unpublished data). This observation is similar to that reported earlier where there was higher tendency of females to develop DHF/DSS [9], [19] with higher mortality rate in females [22] even though males consistently comprised of the larger proportion of both DF and DHF, especially in the ≥15 years age group [22], [23]. More deaths among girls, especially those among the pediatric group, was also reported in Vietnam in 1996–2009, despite the predominance of boys in dengue cases [24], [25]. Currently, there is no satisfactory explanation to this phenomena but there are suggestions that this may be due to the more robust immune response in females, resulting in females to be more prone to develop greater inflammatory response or higher susceptibility to capillary permeability [26], [27]. There was no evidence of differences in the susceptibility and severity to dengue between the different ethnic groups in Malaysia as the percentages of deaths paralleled the ethnic composition of patients who visited UMMC (unpublished data). This is similar to earlier findings done among the fatal cases in Singapore [28], [29].

On average the patients in this review were admitted on day five of illness and most of them had defervesce. This was followed by rapid deterioration of clinical condition. Four patients died within 24 hours of admission and the remaining four died within 5 days of admission. Our observation is consistent with an earlier study done on seven dengue deaths in Singapore where the reported mean period of illness prior to hospitalization was 4.8 days. The mean duration of hospitalization before deaths, however, was longer at 13.7 days [29]. A study in Cuba with 12 fatal cases also reported worsening clinical condition and death occurring at an average of 3.75 and 7.5 days post-hospitalization, respectively [30]. However, the study reported hospitalization of patients at an average of 2.9 days post-onset of illness. This rapid deterioration in the clinical condition is consistent with those presenting with advance stage of disease. Late hospitalization may also be a possible contributing factor to increased risk of mortality and rapid deterioration in severe dengue [24].

Mortality in dengue may also be due to the presence of acquired co-morbidities such as obesity, alcoholism, smoking and the presence of other chronic illnesses such as diabetes mellitus (DM) [31], [32]. Worsening of co-morbidities, rather than directly from dengue infection [29], [32] could be the reason for death seen especially in adults. The presence of DM is especially prominent among our dengue death cases. This observation is consistent with several earlier reports [29], [31], [32] implicating DM, the most common chronic disease in Malaysia especially among females, as a possible contributing factor to death in severe dengue [33]. Asthma, hypertension, chronic obstructive pulmonary disease and chronic renal insufficiency, were other important reported co-morbidities contributing to dengue fatalities [29], [31], [32], [34], [35] but these were not explicitly seen in our study.

Common clinical features of dengue seen in our study include general body ache, abdominal pain, plasma leakage, diarrhea, vomiting, dehydration and bleeding manifestations. Some of the symptoms are consistent with warning signs for severe dengue [3]. Similar findings, especially gastrointestinal symptoms have been reported in other studies [29], [30], [32] emphasizing the importance of warning signs as a tool to recognize patients at risk of severe dengue.

Hepatomegaly, a common and important clinical feature associated with DHF/DSS or severe dengue [36], [37], was seen in four patients; two from physical examination and another two at postmortem. Hepatomegaly may have been an under-diagnosed clinical feature in our study possibly due to the insufficient documentation. The frequency of hepatomegaly may be higher as it was seen in two out of the three postmortem conducted. Hepatosplenomegaly is a clinical feature associated with macrophage activation syndrome (MAS) seen in many autoimmune diseases [38], [39]. It may also be associated with DENV infection [40]. However, splenomegaly was not identified as a clinical feature in our series of patients and was only seen in two patients from their autopsy studies.

At least eight of the fatal cases in our study had evidence of secondary dengue infection, which has been associated with severe outcome of dengue via antibody-dependent enhancement (ADE) [41] and T cell original antigenic sin [42]. All our patients with results available had thrombocytopenia but only five had high hematocrit levels. Platelet count of less than 50×10^9^/L concurrent with hemoconcentration has been shown to increase dengue mortality by six-fold [43]. Three patients (Patient 3, 4 and 7) had normal hematocrit levels despite clear evidence of severe plasma leakage. Patient 4 and 7 had clinical evidence of severe bleeding and Patient 3 may have occult bleeding. This may be the explanation for their ‘normal’ hematocrit levels at presentation. Therefore, hematocrit may not be a sensitive marker of plasma leakage in dengue with severe bleeding. In our study, elevated liver enzymes of >1000 IU/L was common. Equally common was hypoalbuminemia concurrent with elevated liver transaminases. Elevated liver transaminases and hypoalbuminemia could be a good indicator of vascular leakage or hepatic dysfunction in DHF and could be used as a significant marker in identifying cases of severe dengue [44].

Other clinical presentation and severe manifestation of dengue fever in our cohort of patients included involvement of other organs, leading to multi-organ failure. Many patients had acute hepatitis leading to liver impairment and coagulopathy. There was also evidence of tubular necrosis of the kidney, inflammation of the heart, and pulmonary hypertension on postmortem. One patient had cardiogenic shock with evidence of myocarditis, pericarditis, pericardial effusion and global left ventricular hypokinesia. The myocardial injuries could be secondary to the cellular immune response and the production of inflammatory cytokines, or as direct result of DENV infection of the myocardial tissue [45], [46]. These uncommon presentations are often related to poor prognosis and associated with high mortality in severe dengue [47], [48], [49].

In our study, decompensated DSS with evidence of massive plasma leakage, massive bleeding, MOF and coagulopathy were the primary cause of deaths. Per rectum bleeding, vaginal bleeding and gastrointestinal bleeding were the commonest sites of severe bleeding. These observations are consistent with those reported in a number of other studies [30], [50]. Here we also report hemorrhage over the brain hemispheres, the endocardium and septum of the heart and intra-alveolar demonstrated from autopsy. It has been suggested that several of these rare hemorrhagic manifestations has become more apparent and significant in DHF in the past 30 years and carries a higher risk of mortality [49], [50]. The increasing reports of uncommon manifestations in dengue may be reflective of the shift in demographics where there is increasing incidence of DHF or severe dengue among the older age group of patients.

The present study is a retrospective descriptive review. Study limitations include limited documentation of clinical information especially for patients who were brought in dead and those who died within 24 hours. This is also a single-center review involving a relatively small number of fatal cases, which may introduce bias in sample selection. A case control study is needed to determine if those common clinical and laboratory findings seen in our case series are exclusive to fatal cases of severe dengue or seen equally in the non-fatal cases.

In conclusion, our study demonstrates a case series of severe dengue leading to death seen primarily in adult females with secondary dengue infection in Malaysia. The possible contributing role of underlying co-morbidities commonly seen in adults especially diabetes mellitus is highlighted. While most patients with fatal outcomes presented with common clinical and laboratory warning signs of severe dengue seen in all ages, the manifestation of uncommon clinical presentations of dengue is most likely a reflection of a change in the demographic pattern of the population being infected with dengue.

## Supporting Information

Checklist S1 STROBE checklist.
(DOC)
